# Benchmarking large language models for medical education: performance on the clinical laboratory technician qualification examination

**DOI:** 10.3389/fmed.2026.1755983

**Published:** 2026-03-16

**Authors:** Yaqing Wang, Yue Jiang, Wen Jin, Weinan Lin, Yijun Xu, Jiangda Wang, Xiuqing Wang, Zhaoxi Fang

**Affiliations:** 1 The Affiliated Hospital of Shaoxing University, Shaoxing, China; 2 Department of Computer Science and Engineering, Shaoxing University, Shaoxing, China; 3 Institute of Artificial Intelligence, Shaoxing University, Shaoxing, China; 4 Zhejiang-ltaly Joint Laboratory on AI & Materials Medical Technology, Shaoxing People's Hospital, Shaoxing, China; 5 School of Computing, College of Science, Engineering and Technology, The University of South Africa, Florida Campus, Roodepoort, South Africa

**Keywords:** clinical laboratory technologist qualification examination, deepseek, laboratory medicine, large language models, model evaluation

## Abstract

Large language models (LLMs) have shown growing applications in medicine, yet their capabilities in the field of clinical laboratory technology remain underexplored. This study aims to evaluate the performance of LLMs in the Chinese Clinical Laboratory Technologist Qualification Examination (CCLTQE) and provide empirical evidence for their application in laboratory medicine. A dataset containing 1,600 single-choice questions is constructed for the CCLTQE exam. The dataset covers four sections: clinical laboratory fundamentals, other medical knowledge related to clinical laboratory technology, clinical laboratory specialized knowledge, and clinical laboratory professional practice competence. We select 12 LLMs for evaluation, including the DeepSeek, GPT, Llama, Qwen, and Gemma series. Results show that Qwen3-235B achieves the highest overall accuracy (89.93%), followed by DeepSeek-R1 (89.75%) and QwQ-32B (89.22%). This study demonstrates that LLMs optimized for Chinese language and domain-specific content demonstrate outstanding performance in CCLTQE, indicating significant potential for AI-assisted education and practice in laboratory medicine.

## 1 Introduction

Large Language Models (LLMs) are deep-learning-based systems trained on massive text corpora, capable of understanding and generating human language (1–3). Representative models such as GPT (4), DeepSeek (5), and Qwen (6) have achieved remarkable success across diverse applications (7, 8). In medicine, LLMs analyze vast literature, clinical guidelines, and case reports, contributing to diagnostic support, medical education, clinical decision-making, and drug discovery (9–12).

In clinical laboratory medicine, LLMs are applied across scenarios including laboratory data processing and analysis, test report generation and interpretation, diagnostic assistance and decision support, as well as laboratory medicine education (13–15). LLM technology not only reduces laboratory costs but also optimizes workflow processes, enhancing testing quality and efficiency. LLMs can automatically identify and extract critical medical information, such as disease symptoms and treatment methods, from vast text sources, including medical literature and patient records. Additionally, LLMs can extract multidimensional data from unstructured electronic health records, including information on social determinants of health (15). They can monitor laboratory testing processes to identify potential errors or quality issues, thereby enhancing the accuracy and reliability of laboratory results (16). In extracting pathology report information, LLMs outperform traditional natural language processing methods, significantly reducing the time, cost, and errors associated with manual data extraction. For example, one study employed ChatGPT to extract data related to pathological tumors, lymph nodes, overall staging, and histology from over 900 pathology reports of lung cancer and pediatric osteosarcoma, achieving an overall accuracy rate of 89% (17).

However, different LLMs exhibit varying performance across specialized domains. Standardized evaluation is thus necessary to assess their reliability and applicability in medical contexts. Prior studies have examined LLMs' performance in licensing exams (18–20). For instance, in the USMLE exam, GPT-4 reached an 86.1% accuracy, outperforming GPT-3.5's 60.2% (20). In (18), the authors evaluated the performance of ChatGPT in 3 years' worth of the Chinese National Medical Licensing Examination (NMLE). Results showed that ChatGPT's performance was lower than that of the medical students. The authors in Zong et al. (21) proposed a comprehensive evaluation platform to assess LLM performance across multiple medical licensing examinations worldwide, spanning different countries, languages, and examination formats. Their results demonstrated substantial variability among models and highlighted the influence of language background and domain alignment on examination outcomes, underscoring the necessity of context-specific evaluation frameworks. Similarly, Zong et al. (22) conducted a large-scale, multi-year analysis of ChatGPT's performance on several Chinese national medical licensing examinations, revealing that while LLMs exhibit promising capabilities in answering knowledge-based questions, their performance remains inconsistent across specialties and examination types. The study in Lee et al. (23) examined the accuracy of ChatGPT 3.5 on the National Korean Occupational Therapy Licensing Examination. The results showed that ChatGPT could not pass the NKOTLE but demonstrated a high level of agreement between raters. While in Germany's national medical licensing exam, GPT-4 attained 93.1% (24). These studies confirm that advanced LLMs can pass medical licensing exams but still face challenges when handling non-English medical content or country-specific contexts.

While LLMs have been extensively evaluated in general medical contexts, their capabilities in subspecialties such as laboratory medicine remain unclear. Evaluating LLMs in this professional examination helps determine their mastery of specialized knowledge and informs future AI integration in laboratory medicine education and practice. This study systematically evaluates 12 major LLMs–including DeepSeek-R1, GPT-4.1, and Qwen3-235B–on the Chinese Clinical Laboratory Technologist Qualification Examination (CCLTQE). Results show that Qwen3-235B performs best with an overall accuracy rate of 89.93%, followed by DeepSeek-R1 (89.75%) and QwQ-32B (89.22%). Chinese-optimized models demonstrate superior performance across all test domains, achieving an average accuracy 12.79 percentage points higher than other models. Among the 12 tested models, 7 exceed the 80% high-score threshold. These findings confirm the importance of domain-specific training in enhancing model performance on specialized medical examinations, providing crucial insights for applying LLMs in medical education and practice.

## 2 Methods

### 2.1 Dataset construction

This study constructs a question dataset for CCLTQE. This national-level licensing examination assesses healthcare technicians practicing clinical laboratory science in China, serving as the standard assessment to determine whether applicants possess the required professional technical qualifications and competencies. This technical qualification examination aims to scientifically and impartially measure and evaluate medical personnel's professional knowledge, technical skills, and clinical practice capabilities in the field of clinical laboratory science. It ensures that practitioners in this specialty maintain a unified, standardized baseline level of competence, thereby safeguarding healthcare quality and patient safety.

CCLTQE consists of four sections: clinical laboratory fundamentals, other medical knowledge related to clinical laboratory technology, clinical laboratory specialized knowledge, and clinical laboratory professional practice competence. In this work, the question bank was sourced from an educational resource provider specializing in simulation materials for medical licensing examinations in China. The materials were obtained for academic research purposes and consist of simulation questions intended for public educational use. The dataset was structured to reflect the proportional weight and content distribution of the actual examination. To ensure clinical accuracy and relevance, a clinical laboratory specialist reviewed these questions to verify medical correctness and alignment with current practice guidelines.

A complete set of CCLTQE simulation questions was acquired in digital format and organized according to the four official examination sections. Each section contains 400 single-choice questions, totaling 1,600 single-choice questions. These questions cover core areas such as clinical biochemistry, hematology, microbiology, and immunology, comprehensively assessing the theoretical knowledge and practical skills of laboratory medicine professionals. Below is a sample question from the Clinical Laboratory Science section:

*Question:* Which of the following statements regarding cerebrospinal fluid (CSF) protein examination is correct?

A. Turbidimetric method is superior to colorimetric method

B. Pandy's test has high sensitivity and may yield weak positives in normal CSF

C. Protein concentration in neonatal CSF is lower than in adults

D. Normal CSF protein concentration is about 2% of plasma proteins

E. Proin syndrome is a hemorrhagic encephalopathy

*Answer: B*

### 2.2 Models evaluated

After constructing the dataset, we evaluate a diverse range of LLMs to ensure both architectural and linguistic representativeness. The selected models encompass both large, cutting-edge models that excel in natural language processing tasks and small, lightweight models deployable in resource-constrained environments. Balancing cost and performance, we select 12 representative LLMs, including the DeepSeek series, GPT series, Gemma series, and Qwen series. These models represent diverse architectures and scales within current LLM technology, ranging from billions to hundreds of billions of parameters, and encompass bilingual processing capabilities in Chinese and English. Model selection is based on their known performance in medical question-answering tasks, API availability, and diversity in parameter scale to ensure comprehensive and representative evaluation results. Table 1 below displays the basic parameters of these models.

**Table 1:** List of evaluated large language models.

**No**.	**Model**	**Parameters**	**Release date**	**Company**
1	DeepSeek-R1	671B	Jan 2025	DeepSeek
2	DeepSeek-V3Pro	671B	Mar 2025	DeepSeek
3	DeepSeek-R1-32B	32B	Jan 2025	DeepSeek
4	GPT-4o	200B	May 2024	OpenAI
5	GPT-4.1	N/A	Apr 2025	OpenAI
6	GLM-4-32B	32B	Apr 2025	THUDM
7	GLM-Z1-32B	32B	Mar 2025	THUDM
8	Llama4-scout	109B	Apr 2025	Meta
9	Gemma-2-27B	27B	Jun 2024	Google
10	Gemma-3-27B	27B	Mar 2025	Google
11	QwQ-32B	32B	Mar 2025	Alibaba
12	Qwen3-235B	235B	Apr 2025	Alibaba

### 2.3 Testing procedure

We used a standardized prompt to ensure consistency across all models. The prompt template is as follows:

You are a clinical laboratory specialist. Please select the single correct answer from the five options (A, B, C, D, E) for the following question. Provide only the letter of the correct answer.

Question: [Question text]

Options: A. […] B. […] C. […] D. […] E. […]

All evaluated models were accessed through their official public APIs between October and November 2025. Each model was queried using default inference parameters as provided by the respective platforms, including temperature and maximum token length. No manual parameter tuning or model-specific optimization was performed. This unified configuration was adopted to ensure experimental fairness and reproducibility. Responses were programmatically extracted and compared against the ground-truth answer. If a model's output did not contain exactly one of the option letters (A–E), it was recorded as incorrect.

## 3 Results

Table 2 presents the test results for 12 LLMs across four sections of the CCLTQE: clinical laboratory fundamentals (Section I), other medical knowledge related to clinical laboratory technology (Section II), clinical laboratory specialized knowledge (Section III), and clinical laboratory professional practice competence (Section IV).

**Table 2:** Test results.

**Model name**	**Section I (%)**	**Section II (%)**	**Section III (%)**	**Section IV (%)**	**Overall accuracy (%)**
Qwen3-235B	91.75	91.50	89.22	87.25	89.93
DeepSeek-R1	91.75	91.41	85.03	90.79	89.75
QwQ-32B	92.25	88.44	87.97	88.22	89.22
DeepSeek-V3Pro	91.25	88.75	80.75	78.75	84.88
DeepSeek-R1-32B	88.50	86.25	78.00	79.90	83.17
GLM-Z1-32B	88.00	87.75	80.00	75.77	82.91
GPT-4.1	82.25	83.75	80.25	77.94	81.00
GPT-4o	83.00	81.50	79.50	75.50	79.88
Llama4-scout	82.25	79.75	71.75	71.00	76.19
GLM4-32B	84.75	81.25	63.25	63.00	73.06
Gemma3-27B	71.00	67.25	59.75	62.00	65.00
Gemma2-27B	66.50	63.00	47.00	53.50	57.50

The results indicate that in Section I, most models achieved over 85% accuracy. QwQ-32B leads with 92.25% accuracy, while DeepSeek-R1 and Qwen3-235B both exceed 91%. In Section II, Qwen3-235B attains the highest score of 91.50%. Section III proves most challenging for most models, though Qwen3-235B still maintains a high level of 89.22%. In Section IV, both Qwen3-235B and DeepSeek-R1 exceed 87%.

**Figure 1:**
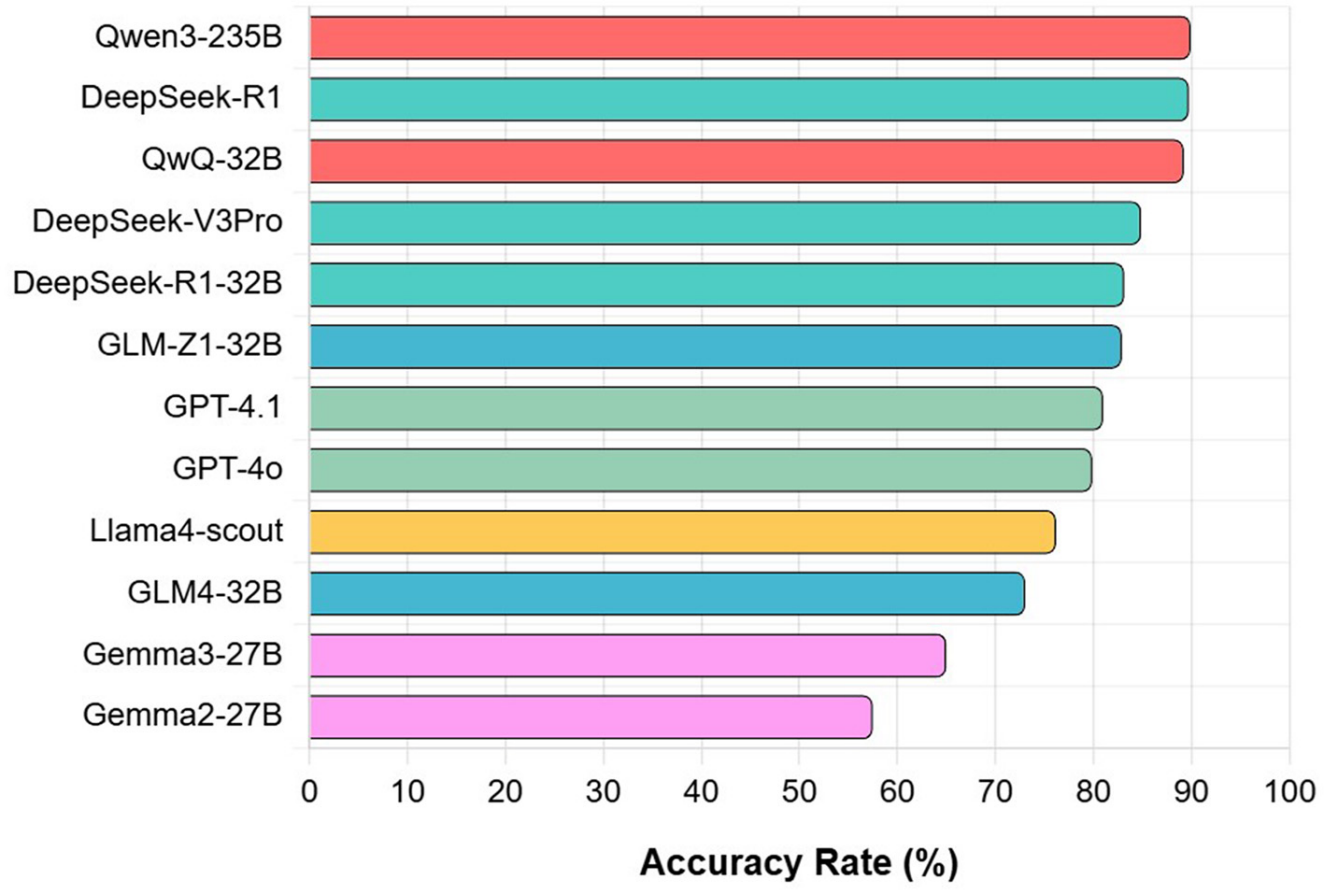
Overall accuracy of 12 LLMs.

Figure 1 shows the overall accuracy of this test. From the figure, it can be seen that the performance of the 12 models varies significantly. Qwen3-235B demonstrates the strongest performance, achieving an overall accuracy of 89.93%, followed closely by DeepSeek-R1 (89.75%) and QwQ-32B (89.22%). GPT-4o and GPT-4.1 achieve accuracy rates of 79.88 and 81.00%, respectively. Models with comparatively lower performance include Gemma3-27B (65.00%) and Gemma2-27B (57.50%). These findings indicate that LLMs trained on Chinese corpora demonstrate superior capabilities in understanding and applying specialized knowledge.

Beyond descriptive accuracy comparisons, we further investigated whether the observed performance differences between models were statistically significant. Since each LLM produced binary outcomes (correct or incorrect) on the same set of examination questions, the resulting predictions constituted paired nominal data. Therefore, pairwise statistical comparisons were conducted using McNemar's test, which is specifically designed to assess differences between two classifiers evaluated on identical samples. We conducted pairwise McNemar tests on the 12 models in the clinical laboratory specialized knowledge assessment. 38 (57.6%) showed significant differences (*p* < 0.05). The Gemma series performed relatively poorly, differing significantly from most models, while the Qwen3 series excelled. Performance tiers emerged: top (Qwen3 series), mid-high (e.g., GLM-4-32B, GPT-4o), and lower (DeepSeek and Gemma series). Notably, within-series scale differences (e.g., Qwen3-235B vs. QwQ-32B) were significant, whereas DeepSeek models were internally consistent. These variations likely stem from architecture, training data, parameters, and domain tuning.

## 4 Discussion

### 4.1 Analysis of core error patterns in the model

Analysis of questions with high error rates in the model's responses revealed certain limitations in the large model's expertise regarding clinical laboratory techniques. Firstly, the model demonstrated fragile recall of precise numerical values and classification criteria. Questions involving specific numerical values, such as the six-type classification of hyperlipoproteinemia and microscopic observation of ten or more fields of view, exhibited the highest error rates. This indicates the model's memory for numerical information lacks stable associations and is susceptible to interference from similar values or outdated standards. Secondly, the model exhibits deficiencies in contextualizing specialized terminology, struggling to grasp precise meanings within specific medical contexts. For instance, confusion regarding antigen specificity in the Witte reaction indicates that the model's comprehension of medical concepts remains confined to superficial lexical levels. Thirdly, the model exhibits a disconnect between clinical practice and theoretical knowledge, lacking the capacity to translate textbook knowledge into clinical judgement. This is exemplified by the confusion between subjective symptoms and objective criteria when determining successful bone marrow aspiration. The underlying causes lie in the scarcity of authoritative professional content within the training data and the inherent limitations of current model architectures in processing high-precision, highly logical specialized knowledge.

### 4.2 Performance comparison and analysis

The superior performance of models such as Qwen3-235B and DeepSeek-R1 can be attributed to several key factors. First, both models have undergone extensive pretraining on large-scale, high-quality Chinese corpora, granting them a strong foundation in understanding the linguistic nuances and domain terminology prevalent in Chinese medical texts. Second, they have benefited from advanced instruction tuning and alignment using biomedical and technical datasets, which enhances their ability to reason about clinical concepts and follow specialized instructions. Third, their substantial parameter scale (235B and 671B, respectively) and incorporation of modern architectural advances, such as reasoning-enhanced training and chain-of-thought optimization, further bolster their capacity for complex clinical problem-solving. While the possibility of prior exposure to similar question patterns cannot be entirely ruled out, these intrinsic strengths collectively explain their high accuracy on the CCLTQE and underscore the importance of language-specific and domain-adapted training in medical LLM applications.

A phenomenon worthy of further investigation was observed during testing: QwQ-32B, with a parameter count of merely 32 billion, outperformed DeepSeek-V3Pro, which boasts a parameter count of 671 billion, in overall performance. This suggests that within the highly specialized domain of medical knowledge, model performance is not solely determined by parameter scale, but more likely depends on a series of targeted optimization strategies. QwQ may have undergone more systematic and in-depth adaptation training within the medical domain, enabling it to grasp medical terminology systems, clinical knowledge structures, and diagnostic logic with greater precision. In contrast, large-scale general-purpose models, while offering broad knowledge coverage, may lack sufficient deep optimization for specialized subfields. Secondly, specialized reinforcement of reasoning capabilities proves crucial. QwQ-32B demonstrates greater stability and logical consistency when handling questions requiring multi-step clinical reasoning, interference exclusion, and negative judgements. This suggests it may have undergone specialized training in chained reasoning and similar cognitive processes.

## 5 Conclusion

This study systematically evaluates 12 LLMs on the CCLTQE examination. Chinese-optimized LLMs, such as Qwen3-235B and DeepSeek-R1, achieved nearly 90% accuracy, substantially surpassing the passing threshold. The findings highlight the importance of domain-specific fine-tuning and provide evidence for integrating LLMs into laboratory medicine education and practice. Future work should explore interactive and reasoning-based evaluation frameworks to better capture the practical capabilities of LLMs in clinical laboratory contexts.

## Data Availability

The original contributions presented in the study are included in the article/supplementary material, further inquiries can be directed to the corresponding author.
